# Measles, Mumps, Rubella Vaccine (PRIORIX): Recommendations of the Advisory Committee on Immunization Practices — United States, 2022

**DOI:** 10.15585/mmwr.mm7146a1

**Published:** 2022-11-18

**Authors:** Elisabeth Krow-Lucal, Mona Marin, Leah Shepersky, Lynn Bahta, Jamie Loehr, Kathleen Dooling

**Affiliations:** ^1^Division of Viral Diseases, National Center for Immunization and Respiratory Diseases, CDC; ^2^Minnesota Department of Health; ^3^Cayuga Family Medicine, Ithaca, New York.

Vaccination is the main means for preventing measles, mumps, and rubella virus infections and their related complications (*1*,*2*). Achieving and maintaining high 2-dose measles, mumps, and rubella vaccination coverage in the United States has led to elimination of endemic measles in 2000, rubella and congenital rubella syndrome in 2004, and a sharp decrease in mumps cases. However, measles and rubella remain endemic in many countries, leading to importations of cases and occasional local transmission within the United States (*3*). Reported U.S. mumps cases declined >99% from the prevaccine period (*4*); however, mumps is endemic worldwide, and since 2006, the number of mumps cases and mumps outbreaks has increased in the United States, with wider geographic spread since 2016 (*4*). Given the risk for importation of measles and rubella and the resurgence of mumps, maintaining high measles, mumps, and rubella (MMR) vaccination coverage is important. Since 1978, only one MMR vaccine, M-M-R II (Merck and Co., Inc.), has been available in the United States. On June 6, 2022, the Food and Drug Administration approved a second MMR vaccine, PRIORIX (GlaxoSmithKline Biologicals), for the prevention of measles, mumps, and rubella in persons aged ≥12 months. The three live attenuated viruses contained in PRIORIX are genetically similar or identical to the corresponding components in M-M-R II (Table) (*5*–*7*). On June 23, 2022, the Advisory Committee on Immunization Practices (ACIP) unanimously recommended PRIORIX as an option to prevent measles, mumps, and rubella according to the existing recommended schedules and for off-label uses (i.e., indications not included in the package insert)* (*1*,*2*). ACIP considered PRIORIX to be safe, immunogenic, and noninferior to M-M-R II. Both PRIORIX and M-M-R II are fully interchangeable for all indications for which MMR vaccination is recommended. This report contains ACIP recommendations specific to PRIORIX and supplements the existing ACIP recommendations for MMR use (*1*,*2*).

**TABLE:** Components and infectious dosage* of measles, mumps, and rubella vaccines^†^ licensed in the United States

Vaccine characteristic	Viral component		
	Measles	Mumps	Rubella
**M-M-R II**			
Strain	Enders' Edmonston	Jeryl Lynn (B level)	Wistar RA 27/3
Infectious dose, minimum and maximum release potencies	≥10^3.0^–10^3.8^ TCID_50_	≥10^4.1^–10^4.8^ TCID_50_	≥10^3.0^–10^3.6^ TCID_50_
**PRIORIX**			
Strain	Schwarz	RIT4385	Wistar RA 27/3
Infectious dose, minimum and maximum release potencies	≥10^3.4^–10^4.5^ CCID_50_	≥10^4.2^–10^5.6^ CCID_50_	≥10^3.3^–10^4.4^ CCID_50_

**Abbreviations**: CCID_50_ = cell culture infectious dose; TCID_50_ = tissue culture infectious dose.

* TCID_50_ and CCID_50_ are closely related measures describing how much infectious virus is contained in a vaccine product.

**^†^** Both the measles and rubella strains in M-M-R II and PRIORIX are 100% identical on a nucleotide level. The Jeryl Lynn strain used in M-M-R II is a mixture of two viral lineages, JL1 and JL2. RIT4385 is a pure clone of JL1 and is 100% identical on a protein level to M-M-R II’s JL1 component.

During January–June 2022, the ACIP Measles, Mumps, and Rubella Vaccine Work Group (Work Group) held monthly conference calls to review and assess the safety and immunogenicity of PRIORIX and to discuss implementation issues. The Work Group identified the following outcomes of interest for evaluation: 1) prevention of measles, mumps, and rubella; 2) short-term humoral immunity; 3) persistence of the humoral immune response; 4) reactogenicity of grade 3 or higher^†^; 5) vaccine-related serious adverse events (SAEs)^§^; and 6) additional adverse events of interest (i.e., rate of febrile seizures, aseptic meningitis, and immune thrombocytopenic purpura [ITP]). SAEs and reactogenicity of grade 3 or higher were evaluated only in studies conducted at or above the licensed U.S. potency for PRIORIX. Additional adverse events and immunogenicity were evaluated at any potency of PRIORIX.^¶^ The Evidence to Recommendations (EtR) framework was used to organize Work Group deliberations.**

Data on the outcomes of interest were summarized based on findings from a systematic review of the literature in PubMed, Medline, Embase, Scopus, Cochrane databases, and clinicaltrials.gov. Search terms for the literature review, study inclusion criteria, and supporting evidence are available online. All studies conducted with PRIORIX at the U.S. potency were included. For studies conducted at a potency different from that for the U.S.-licensed product, the evidence reviewed was restricted to the highest level of evidence: experimental design (i.e., randomized controlled clinical trials) or high-quality reviews (i.e., Cochrane reviews, systematic reviews, or meta-analyses).

The Work Group reviewed all included studies of PRIORIX to assess the safety and immunogenicity of PRIORIX and discussed implementation issues. Summaries of Work Group discussions were presented to ACIP on February 23, 2022 and on June 23, 2022. At the June 2022 meeting, a proposed recommendation was presented to the committee and, after a public comment period, was unanimously approved by the voting ACIP members. PRIORIX is recommended according to the existing recommended schedules and off-label uses as an option to prevent measles, mumps, and rubella.

## Summary of Key Findings

SAEs related to administration of PRIORIX were assessed using findings from four randomized controlled clinical trials at the licensed U.S. potency of PRIORIX and one Cochrane review with PRIORIX at any potency (*8*–*12*). Four additional observational studies and one additional systematic review addressed additional adverse events of interest (i.e., rate of febrile seizures, aseptic meningitis, and ITP) (*13*–*17*). Outcomes for PRIORIX were compared with those for M-M-R II. In the four randomized controlled clinical trials at the U.S. potency of PRIORIX, safety profiles among 1,960 subjects receiving 1 or 2 doses of PRIORIX were compared with those among 933 subjects randomized to receive 1 or 2 doses of M-M-R II. The subjects ranged in age from 12 months to 12 years, with 90% aged 12–15 months. The frequency of vaccine-related SAEs was similar across the vaccine groups: 0.0%–0.2% among subjects receiving PRIORIX and 0.0%–0.3% among those receiving M-M-R II (*8*–*11*). No significant difference in frequency of vaccine-related SAEs was observed within each individual study; pooled estimates were not calculated.

The rate of febrile seizures is highest during the 6–11 days after vaccination for all MMR vaccines and is estimated to be 3.3–8.7 per 10,000 doses, based on two studies conducted in the United Kingdom, which included both PRIORIX and M-M-R II (*13*,*15*). In the clinical trials with PRIORIX of any potency that GlaxoSmithKline Biologicals conducted in the United States, after receipt of a first dose of MMR (PRIORIX or M-M-R II) at age 12–15 months, the rate of febrile seizures attributable to vaccination among 8,386 PRIORIX recipients was 9.5 per 10,000 (95% CI = 4.4–19.6) compared with 14.0 per 10,000 (95% CI = 5.2–34.8) among 3,561 M-M-R II recipients. These studies included coadministration of recommended age-appropriate vaccines, and all found the differences in rates of febrile seizures between the two vaccines to be nonsignificant (*10*,*11*,*18*,*19*). Similarly, the time course of fever was comparable for both vaccines across all studies, with most instances observed 5–12 days postvaccination (Remon Abu-Elyazeed, MD, PhD, GlaxoSmithKline Biologicals, personal communication, March 2022). No evidence of an association of aseptic meningitis with MMR vaccination was reported in the literature for vaccines containing Jeryl Lynn or Jeryl Lynn–derived mumps strains, which are included in both M-M-R II and PRIORIX for immunization against mumps (*12*,*15*,*20*).

ITP is associated with the receipt of live attenuated measles vaccines (*12*,*14*,*16*,*17*). In the four randomized controlled clinical trials at the U.S. potency of PRIORIX, one case of ITP was identified among 1,960 PRIORIX recipients and one case among 933 M-M-R II recipients. From a previous postmarketing study conducted in the United States, the rate of ITP after M-M-R II is estimated at 2.5 per 100,000 doses (*14*). However, strain- or vaccine formulation–specific data on ITP risk are sparse. Based on the clinical trials and the literature (*12*,*14*,*16*,*17*), the rates of ITP after vaccination were considered similar for PRIORIX and M-M-R II.

Short-term humoral immunity was assessed using data from 13 randomized controlled trials (*8*–*11*,*18*,*19*,*21*–*27*), four at the licensed U.S. potency of PRIORIX, and nine at a lower potency of PRIORIX used in other countries. Serologic response thresholds were achieved for all three antigens in all studies. Antibodies in all studies were more than 8.8-fold higher than the predefined seroresponse threshold for measles (200 mIU per mL; correlate of protection 120 mIU per mL) and more than 4.2-fold higher than the rubella correlate (10 IU per mL). Although an antibody correlate of protection has not been established for mumps, the anti-mumps antibody level was ≥3.3-fold higher than the mumps seroconversion threshold (10 IU per mL). The four studies conducted with PRIORIX at the U.S. potency found no significant difference in anti-measles, anti-mumps, or anti-rubella geometric mean concentrations (GMC) after the first dose between PRIORIX and M-M-R II recipients. Among the nine studies at a lower PRIORIX potency, eight showed no statistically significant difference between anti-measles or anti-rubella GMC levels, and seven showed no statistically significant difference between anti-mumps GMC levels. One study reported on persistence of the humoral immune response (2 years after vaccination) and found no difference between vaccines (*8*). None of the four studies that reported on GMC after a second dose noted a significant difference for any antigen at any potency after a second dose between PRIORIX or M-M-R II recipients (*9*,*18*,*21*,*28*).

Additional data reviewed within the EtR framework included findings from a focus group conducted with state immunization managers and a survey of pediatric and general practitioners regarding the feasibility for use and acceptability of PRIORIX. Both the focus group and the survey findings supported the interchangeability of M-M-R II and PRIORIX and the benefit of having a second MMR vaccine option available.

## Rationale for Recommendation

Given the similarities in potency (Table) and vaccine components, and evidence for similar safety and immunogenicity, as well as stakeholder support, PRIORIX and M-M-R II are considered fully interchangeable, including for all off-label recommended uses. Either vaccine may be administered in any situation in which an MMR virus–containing vaccine is indicated. Two interchangeable vaccines from different manufacturers will help safeguard vaccine supply in the United States to maintain measles and rubella elimination and mitigate mumps cases and outbreaks.

## ACIP Recommendation

PRIORIX is recommended according to the existing MMR recommended schedules and off-label uses (*1*,*2*) as an option to prevent measles, mumps, and rubella.

## Clinical Guidance

PRIORIX is supplied as a single-dose vial of lyophilized antigen to be reconstituted with the accompanying prefilled syringe of sterile water diluent. A single dose after reconstitution is approximately 0.5 mL. PRIORIX is formulated without preservatives and is administered as subcutaneous injection (the same as M-M-R II) (*5*,*29*).

PRIORIX may be used according to the existing MMR recommendations for both on- and off-label use for prevention of measles, mumps, and rubella^††^ (*1*,*2*). For routine vaccination, 2 doses are recommended, the first at age 12–15 months, and the second at age 4–6 years. For catch-up vaccination of previously unvaccinated children and adolescents, 2 doses should be administered ≥4 weeks apart. Before international travel, infants aged 6–11 months should receive a single dose. Travelers aged ≥12 months who have not received 2 doses of MMR should receive 2 doses separated by ≥28 days.

During a measles outbreak, infants aged 6–11 months should receive a single dose of MMR. For measles postexposure prophylaxis in unvaccinated persons, 1 dose of MMR should be administered within 72 hours of exposure to a person with infectious measles, and the 2-dose series (i.e., the second of 2 MMR doses) should be completed ≥28 days later. During mumps outbreaks, a third dose of MMR is recommended for persons identified by public health authorities as being part of a group or population at increased risk for acquiring mumps because of an outbreak.

## Interchangeability

PRIORIX and M-M-R II are fully interchangeable. ACIP General Best Practices states a preference that doses of vaccine in a series come from the same manufacturer; however, vaccination should not be deferred when the manufacturer of the previously administered vaccine is unknown or when the vaccine from the same manufacturer is unavailable (*30*). Studies have shown that PRIORIX is safe and immunogenic when administered as a second dose after M-M-R II (*10*,*21*).

## Timing of Vaccination and Coadministration with Other Vaccines

PRIORIX can be administered concomitantly, at different anatomic sites, with other routine childhood vaccines. Concomitant administration of PRIORIX with other live and nonlive vaccines^§§^ has been studied; results indicated no safety concerns or evidence for interference in the immune response to either (*8*,*10*,*11*,*18*,*19*,*21*,*28*). Additional live virus vaccines not administered on the same day should be separated by ≥4 weeks (*30*).

## Precautions and Contraindications

Before administering PRIORIX, health care providers should consult the package insert for precautions, warnings, and contraindications (*5*,*29*). Contraindications for PRIORIX are the same as those for M-M-R II. PRIORIX should not be administered to persons with a history of severe allergic reactions (e.g., anaphylaxis) to any component of the vaccine or after a previous dose of any measles, mumps, and rubella virus–containing vaccine (unlike M-M-R II, PRIORIX does not contain gelatin); persons with severe humoral or cellular (primary or acquired) immunodeficiency; or women who are pregnant. Pregnancy should be avoided for 1 month after receipt of MMR. Additional information on warnings and precautions can be found in the package insert and previous vaccine recommendations (*1*,*5*,*29*).

## Reporting of Vaccine Adverse Events

Adverse events following administration of any vaccine should be reported to the Vaccine Adverse Event Reporting System (VAERS). Reports can be submitted to VAERS online, by fax, or by mail. Additional information about VAERS is available by telephone (1-800-822-7967) or online (https://vaers.hhs.gov). Any future revisions to this ACIP recommendation will be dictated by reported adverse events and new research evidence.

**Summary**

**What is already known about this topic?**

Since 1978, M-M-R II has been the only measles, mumps, and rubella (MMR) combination vaccine used in the United States. In June 2022, the Food and Drug Administration licensed an additional MMR vaccine, PRIORIX.

**What is added by this report?**

The Advisory Committee on Immunization Practices recommends PRIORIX as an additional option to prevent MMR according to existing vaccine recommendations and off-label uses.

**What are the implications for public health practice?**

Both vaccines are interchangeable for all indications for which MMR vaccination is recommended. Availability from multiple manufacturers safeguards U.S. vaccine supply.

## Footnotes

* Off-label uses for both M-M-R II and PRIORIX: infants aged 6–11 months who will travel or live abroad or during measles outbreaks and third dose of MMR in persons previously vaccinated with 2 doses of a mumps virus–containing vaccine who are identified by public health authorities as being part of a group or population at increased risk for acquiring mumps because of an outbreak. In addition, PRIORIX is indicated for off-label use for measles postexposure prophylaxis; M-M-R II is not.

† Grade 3 intensity was defined as crying when the limb was moved or the limb was spontaneously painful (pain), event preventing normal activity (drowsiness), crying inconsolably, preventing normal activity (irritability), or not eating at all (loss of appetite).

§ A serious adverse event is defined as an undesirable experience associated with the vaccine resulting in death, hospitalization, or disability or requiring medical or surgical intervention to prevent a serious outcome.

¶ PRIORIX has been licensed outside of the United States since 1997 and has been approved in more than 100 countries at the following potency: measles virus (Enders’ Edmonston strain) ≥10^3.0^cell culture infectious dose_50_, mumps (Jeryl Lynn [B level]) strain ≥10^3.7^cell culture infectious dose_50_, and rubella (Wistar RA 27/3) ≥10^3.0^cell culture infectious dose_50_.

**
https://www.cdc.gov/vaccines/acip/recs/grade/mmr-PRIORIX-etr.html

†† No direct evidence for PRIORIX for off-label uses; recommendation is based on existing ACIP recommendations and comparative use of M-M-R II in similar situations.

§§ Among children aged 12–15 months: with 13-valent pneumococcal conjugate vaccine (PCV13-Prevnar), Varivax (VAR), Havrix (HAV), and 7-valent pneumococcal conjugate vaccine (PCV7). Among children aged 4–6 years: with Kinrix (DTaP-IPV) and Varivax.
