# *Erratum:* Vol. 71, No. 46

**DOI:** 10.15585/mmwr.mm7201a3

**Published:** 2023-01-06

In the report “Measles, Mumps, Rubella Vaccine (PRIORIX): Recommendations of the Advisory Committee on Immunization Practices — United States, 2022,” on page 1465, the second sentence of the first footnote should have read, “In addition, **measles postexposure prophylaxis is an off-label indicated use for PRIORIX; measles postexposure prophylaxis is an on-label indicated use for M-M-R II**.”

